# Metacognition for Spelling in Higher Education Students with Dyslexia: Is There Evidence for the Dual Burden Hypothesis?

**DOI:** 10.1371/journal.pone.0106550

**Published:** 2014-09-05

**Authors:** Wim Tops, Maaike Callens, Annemie Desoete, Michaël Stevens, Marc Brysbaert

**Affiliations:** 1 Ghent University, Ghent, Belgium; 2 University of Groningen, Groningen, The Netherlands; Cardiff University, United Kingdom

## Abstract

We examined whether academic and professional bachelor students with dyslexia are able to compensate for their spelling deficits with metacognitive experience. Previous research suggested that students with dyslexia may suffer from a dual burden. Not only do they perform worse on spelling but in addition they are not as fully aware of their difficulties as their peers without dyslexia. According to some authors, this is the result of a worse feeling of confidence, which can be considered as a form of metacognition (metacognitive experience). We tried to isolate this metacognitive experience by asking 100 students with dyslexia and 100 matched control students to rate their feeling of confidence in a word spelling task and a proofreading task. Next, we used Signal Detection Analysis to disentangle the effects of proficiency and criterion setting. We found that students with dyslexia showed lower proficiencies but not suboptimal response biases. They were as good at deciding when they could be confident or not as their peers without dyslexia. They just had more cases in which their spelling was wrong. We conclude that the feeling of confidence in our students with dyslexia is as good as in their peers without dyslexia. These findings go against the Dual Burden theory (Krüger & Dunning, 1999), which assumes that people with a skills problem suffer twice as a result of insufficiently developed metacognitive competence. As a result, there is no gain to be expected from extra training of this metacognitive experience in higher education students with dyslexia.

## Introduction

Getting a degree in higher education depends on three groups of variables: adequate intellectual abilities, will to work (achievement motivation), and knowing how to study. The last component, on which we will focus, is usually referred to as metacognition [1], [2], [3]. Metacognition was originally defined as cognition about cognition [3], [4]. Gradually, it became clear that metacognition involved more aspects. Efklides [2], [5], [6], [7], for instance, distinguished three components of metacognition: metacognitive knowledge, metacognitive skills, and metacognitive experiences.

Metacognitive knowledge refers to beliefs about cognition stored in long term memory. Metacognitive skills deal with the regulation of the cognitive processes needed for good performance. They include (appropriate) effort allocation, time allocation, planning, executing the various steps towards the goal, checking the progress, adapting the modus operandi if necessary, and evaluating the outcome, so that lessons can be learned for future performance. Metacognitive experiences are based on previous involvements with the task at hand (or related learning conditions). They enable the learner to be better aware of the progress made [6] and to make use of alternative metacognitive skills if needed. They include memories of previous experiences with the task, estimates of efforts and time required for successful performance, feeling of difficulty of the task, and feeling of confidence in one's own abilities.

Much research has confirmed the contribution of metacognition to successful learning e.g., [8], [9], [10]. There is also good evidence that metacognition is involved in the acquisition of adequate reading and writing skills [11], [12], [13], [14], [15]. Metacognition therefore may be an important factor in understanding why some people struggle with reading and/or writing [16], [17], [18], [19]. These people are commonly referred to as suffering from dyslexia.

Dyslexia is a specific learning disorder characterized by a persistent problem in learning to read and/or write words or in the automatization of the reading and writing process (Dyslexia Foundation Netherlands; [20]). The level of reading and/or writing is significantly lower than what can be expected on the basis of the educational level and age of the individual. In addition, the impairment is resistant to remedial teaching (defined as meeting the requirements of the “response to instruction” model; [21]), and the reading and writing deficit cannot be attributed to external and/or individual factors such as socio-economic status, cultural background or intelligence.

The term dyslexia is no longer used in the DSM-5 [22], where it is called a specific learning disorder with impairment in reading (word reading fluency and/or accuracy, reading comprehension) and/or written expression (spelling, grammar and punctuation accuracy, organization of written expression). Still, we will continue to use the term throughout the present study, as we have followed the criteria for dyslexia in the selection of participants, as set out by the Dyslexia Foundation Netherlands.

There is evidence for a genetic component in dyslexia (e.g., [23], [24]), but the prevalence of the problem also depends on environmental features. For instance, it has been reported that dyslexia is more common in languages with opaque letter-to-sound mappings (such as English) than in languages with simple mappings, such as Italian [25] and Welsh [26]. Beginning readers of languages with inconsistent mappings need more time to reach ceiling performance and, apparently, have higher chances of not getting there. Next to the reading and/or spelling impairment, individuals with dyslexia have been reported to show specific (working) memory problems [27], attentional deficits [28], reduced processing speed [29], problems with fast lexical retrieval and arithmetic [30], [31], and less elaborated vocabulary skills [32].

Studies on dyslexia and metacognition come to inconclusive results. Kirby, Silvestri, Allingham, Parrila and Lafave [33] showed that students with dyslexia compensate for their reading difficulties by metacognition. Other studies on metacognition and dyslexia have suggested less sophisticated metacognition in students with dyslexia [16], [34]. Job and Klassen [35] found that adolescents with dyslexia were less accurate at predicting their performance. They overestimated their ability on a spelling and ball-throwing task, with a decrease in accurate performance prediction as the difficulty level increased. The authors called this ‘optimistic miscalibration’. Mason and Mason [36] reported that adult college students with dyslexia showed deficits in metacognitive skills, resulting in problems with selecting and using effective cognitive strategies.

Kruger and Dunning [37], [38] introduced the Dual Burden hypothesis to indicate how people with a skills problem may suffer twice as a result of insufficiently developed metacognitive competence. Not only do these people reach erroneous conclusions and make wrong choices, but their lack of correct metacognitive experiences also hinders them from realising it. Applied to spelling, the knowledge underlying the ability to write without errors is also the knowledge needed to make correct estimates about one's spelling efficiency. Sideridis, Morgan, Botsas, Padeliadu and Fuchs [39] reported some evidence for this possibility, as they observed that knowledge about how to monitor and control one's learning was one of the best predictors of performance in students with dyslexia. Similarly, Trainin and Swanson [40] argued that successful college students with dyslexia have compensated for their cognitive difficulties and processing deficits by relying on metacognition (learning tricks and strategies to cope with the problem, seeking help in time, etc.). Indeed, metacognitive processes (in particular, experiences) make the person aware of the problem and trigger control processes that can serve to help reach the goal that is pursued.

In a recently published Dutch assessment battery for dyslexia in (young) adults [41], three subtests include a measure of metacognition. Two of them are related to spelling. The third applies to morphology and syntax and will not be taken into account in this study because it is not a core deficit in dyslexia. The first spelling test involves a word dictation task, in which participants have to write down spoken words. The second is a proofreading task in which participants have to indicate whether words are correctly written or not. In addition to the primary task, participants also have to indicate how sure they are about their answer (uncertain, almost certain and very certain).

When considering traditional statistical analyses for metacognition in spelling, we saw ourselves confronted with two challenging statistical issues. First, one has to be careful interpreting significant interaction effects in the presence of main effects [42], because a difference between 100 and 120 may be of a similar magnitude as a difference between 10 and 12 (i.e., an increase of 20%). Second, these kinds of analyses often fail to make a clear distinction between performance and metacognition. A proper measure of feeling of confidence or FOC must take into account the difference in skills-levels between the groups. It is easy for people who rarely make spelling errors to feel confident about their performance. Similarly, the best strategy for someone who makes lots of spelling mistakes may be to feel unsure on each trial.

**Figure 1:**
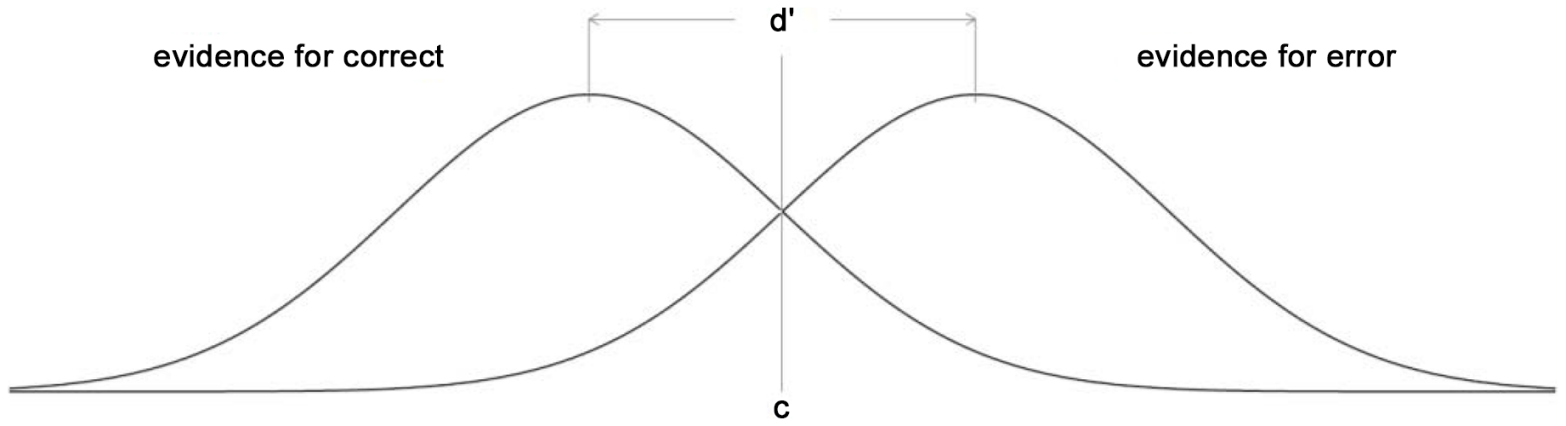
Logic of a signal detection analysis for spelling errors. Both correct spellings and wrong spellings induce a certain degree of evidence for a potential error. This evidence is not fixed but varies from trial to trial as a function of the noise in the system. As a result, there is a normal distribution of evidence when the spelling is correct and a normal distribution of evidence when the spelling is wrong. For someone with good spelling skills, both distributions are far apart, so that there is virtually no overlap between them. This is estimated by the distance d′. We can expect that d′ will be considerably larger for controls than for dyslexics. The second variable of interest is c, the place where the decision criterion is placed. When c is at the intersection of the two distributions (as in the figure), it is positioned optimally, because then the smallest number of errors overall is made. In that case, c = 0. When c is lower than 0, the participant makes too many error judgments (i.e., does so for too many words with correct spellings). Conversely, when c is higher than 0, the participant fails to see too many wrong spellings. The degree of deviation in c from 0 is therefore a good indication of the dual burden theory.

The most elegant way to analyze decision strategies is to make use of signal detection theory, as this nicely separates response criteria from the sensitivity to spelling errors. Figure 1 shows the underlying logic.

Recent advances in statistical methods have made signal detection analysis possible for many more designs than before. DeCarlo [43] showed that the signal detection model can be formulated as a subclass of the generalized linear model. With only one participant and two possible responses, the classical signal detection model can be estimated using a probit regression model with response (e.g., a word is spelled correctly or not) as the dependent variable and type of trial as the predictor (e.g., correct spelling vs. wrong spelling; preferably coded as -0.5 and +0.5). In that case the slope of the regression line corresponds to the distance *d′* and the intercept agrees with the criterion *c*.

According to signal detection theory, both correctly and wrongly spelled words trigger a certain amount of evidence for a spelling error. Because of noise in the system, the degree of evidence triggered by correct and wrong spellings is not always exactly the same, but forms two normal distributions (one for correct spellings and one for wrong spellings). In a proficient speller the degree of error provoked by a correctly spelled word is much lower than the degree of error provoked by a wrongly spelled word. So, it will be easy to make a distinction between both distributions. In contrast, for a poor speller, d′ will be small and there will be a large overlap between the distributions. So, the distance (d′) between the two distributions is an indication of spelling proficiency. This distance is thought to be stable per participant: Individuals cannot manipulate their ability to detect errors.

The second aspect influencing performance is where the response criterion (c) is placed. The total number of judgment errors is minimal when c is placed at the intersection of the two distributions (as shown in Figure 1). This is true for all values of d′. Such optimal performance is called c = 0. When c is lower than zero, then the participant has a tendency to judge too many spellings wrong, including a large number of correct spellings. Conversely, when c is set above 0, the participant has a tendency to report too many spellings as correct, including a disproportionately large number of wrong spellings. Within signal detection theory, it is assumed that participants control the position of c as a function of the perceived costs and benefits of the choice alternatives.

In the present study we wanted to investigate whether Kruger and Dunning's [38] dual burden hypothesis applies to the spelling problems experienced by students entering higher education with an assessment of dyslexia. In particular, we wondered whether students with dyslexia would perform less well on spelling because they lack insight into when they are doing well and when not. This metacognitive experience can be measured with Feeling of Confidence (FOC) judgments. If people have no knowledge of their performance, they feel equally confident (or hesitant) on correct and incorrect trials. In contrast, if they have good knowledge, there will be a significant distinction between correct and incorrect trials.

It is easy to see how the position of c translates into the Dual Burden theory. Not only would students with dyslexia have a smaller value of d′. In addition, they would position the response criteria at a non-optimal place, so that they either fail to recognize the correct spellings they are capable to discriminate (when c<0) or fail to see the spelling errors that are within their reach (when c>0). Deviations of c from 0 indicate that the person has a suboptimal *Feeling of Confidence* (FOC).

## Methods

The study was part of a larger research program in which we also looked at the cognitive profile of the students [29] and their personality profiles [44]. This study was approved by the ethical comity of Ghent University, meaning that the researchers followed the ethical protocol of the university. Students were paid for their participation. All students gave written informed consent and were informed that they could stop at any time if they felt they were treated incorrectly.

### Participants

Two hundred first-year undergraduate students of higher education participated in this study. All students had graduated from secondary school and were in their first year of a professional bachelor (in colleges for higher education) leading to a professional bachelor degree (after three years of education) or an academic bachelor (in some colleges for higher education and in university, preparing for a master degree) in the surroundings of Ghent (one of the main cities in the Northern, Dutch-speaking half of Belgium). The group consisted of 100 students diagnosed with dyslexia and a control group of 100 students without dyslexia or other known neurological or functional disorders (ADHD, ASD, …). All had normal or corrected-to-normal vision and were native speakers of Dutch. Dutch is a language with a more transparent letter-sound-mapping than English, but less transparent than Spanish or Italian (e.g., [45]).

All students with dyslexia who applied for special facilities at the local support office (vzw Cursief) in the academic year 2009–2010 were asked to participate in the study until we had a total of one hundred. To find a group of 100 participants with dyslexia who completed the full study, we had to approach an initial cohort of some 120 students. Of these 120 students a small number of students chose not to cooperate once the study was explained to them. A few more students were lost because they failed to show up at appointments. The students with dyslexia had been diagnosed prior to our study by trained diagnosticians in accordance with the definition of SDN (Stichting Dyslexie Nederland [Foundation Dyslexia Netherlands], [20]). They all met the three criteria of the SDN at the moment of their participation. First, their reading and/or writing skills were significantly lower than could be expected given the age and the educational level of the students. Second, all students met the criterion “resistance to instruction” implying that they had attended remedial programs and received individual tutoring in primary or secondary education for a period of minimum 6 months by either a speech-therapist or a remedial teacher. Third, the reading and/or writing impairment could not be attributed to external or individual factors such as cultural background, intelligence or socio-economic status.

To reflect the inflow in the first year of higher education as much as possible and to construct homogenous groups, matching criteria for recruitment of the control students were restricted to field of study, gender and age. To recruit the control students we used different methods. We asked the students with dyslexia for several names of fellow classmates who would be interested in participating. Amongst these names we selected someone at random. If the student with dyslexia failed to give names (which was the case for about half of the participants), we recruited them ourselves by means of electronic platforms or the guidance counselors at the institution in question. There was no difference between the two groups in socio-economical level based on the educational level of the mother, *χ* ^2^(3) = 4.855, *p* = .183 or father, *χ* ^2^(3) = 2.634, *p* = .452. Educational levels were: lower secondary education, higher secondary education, or post-secondary education either at university or non-university college, as can be seen in Table 1.

**Table 1:** General Information About the Student Groups With and Without Dyslexia.

		Students without Dyslexia N M (*SD*)	Students with dyslexia N M (*SD*)	Effect size Cohen's *d*
Gender	Male	46	46	
	Female	54	54	
Studies	University	66	66	
	College for higher education	34	34	
Age		19.40 *(1.00)*	19.11 *(0.70)*	NA
Fluid IQ		106.80 *(10.80)*	105.40 *(11.00)*	0.13
Word reading		100.40 *(10.60)*	77.00 *(14.20)*	1.97*
Pseudoword reading		59.70 *(13.10)*	40.90 *(10.50)*	1.59*
Word spelling		24.60 *(2.80)*	17.50 *(4.00)*	2.05*

*Note*. * p<.01; NA  =  not applicable; Fluid IQ  =  Total IQ score on the fluid subtests of the KAIT [48]; Word reading  =  Dutch word reading, number of words read correctly in 1 minute time (EMT [46]); Pseudoword reading  =  number of pseudowords read correctly in 1 minute time (de Klepel [47]); word spelling  =  number of words spelled correctly in a word dictation task (GL&SCHR [41]). Effect sizes calculated according to Cohen's *d* (positive d-values represent better performance of the controls and negative values better performance of the students with dyslexia).

Table 1 also shows the results of three literacy tests and one IQ-test we administered to the participants. The verbal tests were word reading (EMT; [46]), pseudoword reading (de Klepel [47];), and word spelling (GL&SCHR; [41]). On all three tests the control group obtained scores within the normal range, whereas the students with dyslexia on average had scores more than 1.5 standard deviations below this level (see the effect sizes in Table 1). The mean fluid IQ measured with the *Kaufman Adolescent and Adult Intelligence Test, Dutch version* [48] was 107 (*SD = *10.8) for the control group and 105 (*SD = *11.0) for the students with dyslexia. The difference in intelligence was not significant, *F* (1, 198) = 0.84; *p* = .36.

### Test description

For this study two subtests of the *Test for Advanced Reading and Spelling* (Test voor Gevorderd Lezen en Schrijven), also called the GL&SCHR [41] were used. The GL&SCHR is a test battery for the diagnosis of dyslexia in Dutch-speaking (young) adults. The two subtests we analyzed measured word spelling and the ability to detect mistakes in sentences (also known as proofreading). We selected these tests because spelling is one of the major problems in adults with dyslexia [29], is important in (higher) education, and is easy to score unambiguously. Guttman split half correlations of the subtests were .69 and .80.

In the first subtest, *Word spelling*, participants had to write 30 words. Half of the words were rule-based (i.e., their spelling required the correct application of the Dutch phoneme-grapheme and morphosyntactic spelling rules); the other half were memory-based words involving inconsistent sound-letter mappings that must be memorized (mostly because the word is a loan word from another language). The test was computer paced. Each participant was given a blue pen and instructed to put on headphones. Words were presented auditorily with a regular interval of 3 seconds, so that students had to produce an immediate response (as in note taking during lectures). After the first hearing, the headphones were put aside and the participant was given a green pen. The student was allowed to use this to correct any mistakes. In addition, the words the student had missed were read out again by the test administrator and the participant used the green pen to write them down. Besides the word that needed to be written, the response form also contained a three point Likert scale to be completed for each word. For each word the participants were asked how sure they were about their spelling by marking one of these three options (certainly correct, rather certain the spelling is correct, or uncertain). There are three scores for this subtest: one for the number of correct responses, one for the total number of words written during the dictation itself (this variable is beyond the scope of the present paper and is, therefore, not further discussed), and one weighted score in which the certainty per item is taken into account. The last scoring is considered as a measure of metacognitive experience, more precisely the Feeling of Confidence or FOC (for more information, see [2]).

The second subtest, *Spelling rules*, was a proofreading task in which participants had to correct spelling mistakes in 20 words or sentences with a misspelled target word. Participants were asked to correct any misspellings in the words or sentences they noticed (the instructions did not say that each word or sentence contained one spelling error on the target word). As was the case in the previous subtest, students rated how certain they felt about their spelling corrections (certainly correct, rather certain correct, uncertain). There are two scores: one based on the number of well corrected target words (independent of the responses to the other words), and one defined as a weighted score (FOC score). The scores were limited to the target words (i.e., a score of zero was given when participants wrongly corrected a correctly spelled filler word).

For each spelling test, there were two scores: (1) the number of correct responses, and (2) the FOC weighted responses. The latter was implemented by the authors of the GL&SCHR as follows for the spelling test. If the students were very certain and they spelled the word correctly, they received five points. If they were almost certain and the word was spelled correctly they received four points. If they were not certain and the spelling was correct, they were given three points. If they were not certain and the spelling was incorrect, they were given two points. If they were almost certain and the spelling was incorrect, they received one point. And finally, if they were very certain about their spelling but it was incorrect, they received zero points.

A similar FOC weighted coding scheme was used for the proofreading task. If the students corrected the error in the target word well and they were very certain, they received five points. If they corrected the target word well but felt less certain, they obtained four points. If they corrected the target word but felt uncertain, they got three points. If they failed to correct the target word or misspelled it while correcting, they got two points if they felt uncertain, one point if the felt almost certain, and zero points if they felt very certain.

### Procedure

The two subtests of the GL&SCHR were part of a larger protocol [29], [49]. The complete test battery involved additional tests such as an intelligence test, reading and spelling tests, a study strategies inventory, a personality test and a semi-structured interview about the socio-emotional and school functioning of the student. The test protocol was split in two counterbalanced parts of about 3 hours each. The order of the tests in part one and part two was determined in such a way that two similar tests were never administered in the same part. There was a break halfway in each session. If necessary, students could take additional breaks. The students with dyslexia started with part one or two according to an AB-design. Their matched control student always started with the same part. The GL&SCHR was taken as a whole in part 2. All tests were administered individually by one of three test administrators (the two first authors and a test psychologist) according to the manual guidelines. After going through the manual guidelines, they observed each other during the first ten test sessions. Afterwards, there was always a brief discussion to key the test administrations as far as possible to one another. Students were tested in a quiet room with the test administrator seated in front of them.

### Signal Detection Theory Analysis

Our data fit within the framework of SDT as described in the introduction, but we need to extend it a little bit. First, we do not have a binary decision, but a three-level rating response (certainly correct, rather certain, uncertain). This can be accommodated by using an ordinal probit regression model instead of the binary probit regression model [43]. In that case we still have one estimate for d′ (the slope), but two intercepts: One that corresponds to the first response criterion c1 (between certainly correct and rather certain correct), and one that corresponds to the second criterion c2 (between rather certain correct and uncertain).

Second, we have 200 participants instead of just one participant. This violates the independence assumption of the linear model, so that we have to use a linear mixed effects model, with additional random intercepts and slopes per participant.

Finally, we want to compare students with dyslexia and control subjects. This can be done by adding an extra predictor (Group) to the model. A main effect of Group then indicates that the two groups used different response criteria. An interaction of Group with Type of trial (the word is spelled correctly or not) points to a difference in the ability to discriminate between trials with and without errors.

The analyses were based on the clmm function from the R package Ordinal [50] (see the Supplemental materials). The formula of the model was as follows: 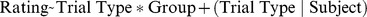



The rating had three levels (certainly correct, rather certain correct, uncertain), so we had an intercept for the transition between certainly correct and rather certain correct, and a second intercept for the transition between rather certain correct and uncertain. Trial Type was contrast coded: the variable was set to −0.5 for ‘correct’ trials and +0.5 for ‘wrong’ trials. By doing this, the intercepts (response criteria) are counted relative to the point where the ‘correct’ and ‘wrong’ distributions cross (i.e., where c = 0).

## Results

First, we analyzed the scores as recommended by the authors of the test. For each test, we had two scores: (1) the number of correct responses, and (2) the FOC weighted responses. Assuming that students with dyslexia are poorer in metacognition than their peers, we expected the difference between both groups to be larger for the FOC weighted scores. As can be seen in Table 2, there was some mixed evidence that the recommended FOC weighted scores resulted in a larger effect size than the percentages of correct responses. There was a difference in the expected direction for the proofreading task but not for the dictation task.

**Table 2:** Performances of Students with Dyslexia on Spelling in Comparison With their Non-dyslexic Peers.

	Students with dyslexia		Students without dyslexia		Cohen's *d*			*p*
	M	*(SD)*	M	*(SD)*	*d*	lower CI	upper CI	
Word Spelling								
*Weighted score (FOC)*	91.59	*(15.87)*	121.40	*(12.84)*	2.06	1.64	2.48	**
*Number correct words*	17.49	*(4.02)*	24.60	*(2.81)*	2.05	1.63	2.47	**
Proofreading								
*Weighted score (FOC)*	50.83	*(11.74)*	62.45	*(13.59)*	0.92	0.49	1.33	**
*Number correct words*	10.05	*(4.87)*	13.81	*(8.15)*	0.56	0.14	0.98	**

Traditional analysis of the FOC weighted scores.

*Note*. p<.05; **p<.01; GL&SCHR  =  Dutch reading and writing test battery for (young) adults [41].

For the reasons outlined in the introduction, we had serious doubts about the usefulness of analyses of variance based on weighted scores. Table 3 shows the outcome of a more promising technique, the SDT analysis, separately for the word dictation task and the proofreading task. The effect of trial type is an estimate of d′ (the distance between the correct and error distribution), the first intercept is an estimate of c1 (response criterion between certainly correct and rather certainly correct), the second intercept is an estimate of c2 (response criterion between rather certainly correct and uncertain).

**Table 3:** Results of the Signal Detection Theory Analysis for the Word Dictation and Proofreading tasks.

		d′	c1	c2
Word spelling	Control	1.03 (z = 13.31, p<.001)	0.12 (z = 3.27, p<.001)	1.13 (z = 29.15, p<.001)
	Dyslexia	0.71 (z = 10.54, p<.001)	−0.08 (z = −2.73, p<.001)	0.93 (z = 27.8, p<.001)
	*Difference*	0.32 (z = 3.13, p<.001)	−0.20 (z = −4.37, p<.001)	
Proofreading	Control	1.02 (z = 10.35, p<.001)	−0.32 (z = −8.15, p<.001)	0.94 (z = 22.39, p<.001)
	Dyslexia	0.84 (z = 8.70, p<.001)	−0.42 (z = −10.76, p<.001)	0.85 (z = 20.74, p<.001)
	*Difference*	0.17 (z = 1.29, p = .197)	−0.09 (z = −1.78, p = .074)	

*Note.* d′ refers to the distance between the two normal distributions (Figure 1), c1  =  the criterion between.

Certainly correct and rather certainly correct, c2  =  the criterion between rather certainly correct and uncertain.

The proportional odds assumption claims that the difference for c1 and c2 is identical.

As expected, d′ was larger for the controls than for the students with dyslexia. This simply refers to their (stable) differences in spelling proficiency. In line with Table 2, the disadvantage of the dyslexic students was larger for the word dictation task than for the proofreading task. More importantly for testing the dual burden theory, both students with dyslexia and control students had their c1 values close to 0 (i.e., the optimal performance level) in the dictation task. This is the criterion between being certain that the spelling is correct and being rather certain that the spelling is correct. The control group tended to be more confident (missing some of the errors they made) than the dyslexic group (thinking some of the correct spellings they made were almost certainly wrong). However, it is not the case that the dyslexic group performed less well than the control group (i.e., the absolute deviation of c1 from 0 was not larger for the dyslexics than for the controls).

**Figure 2:**
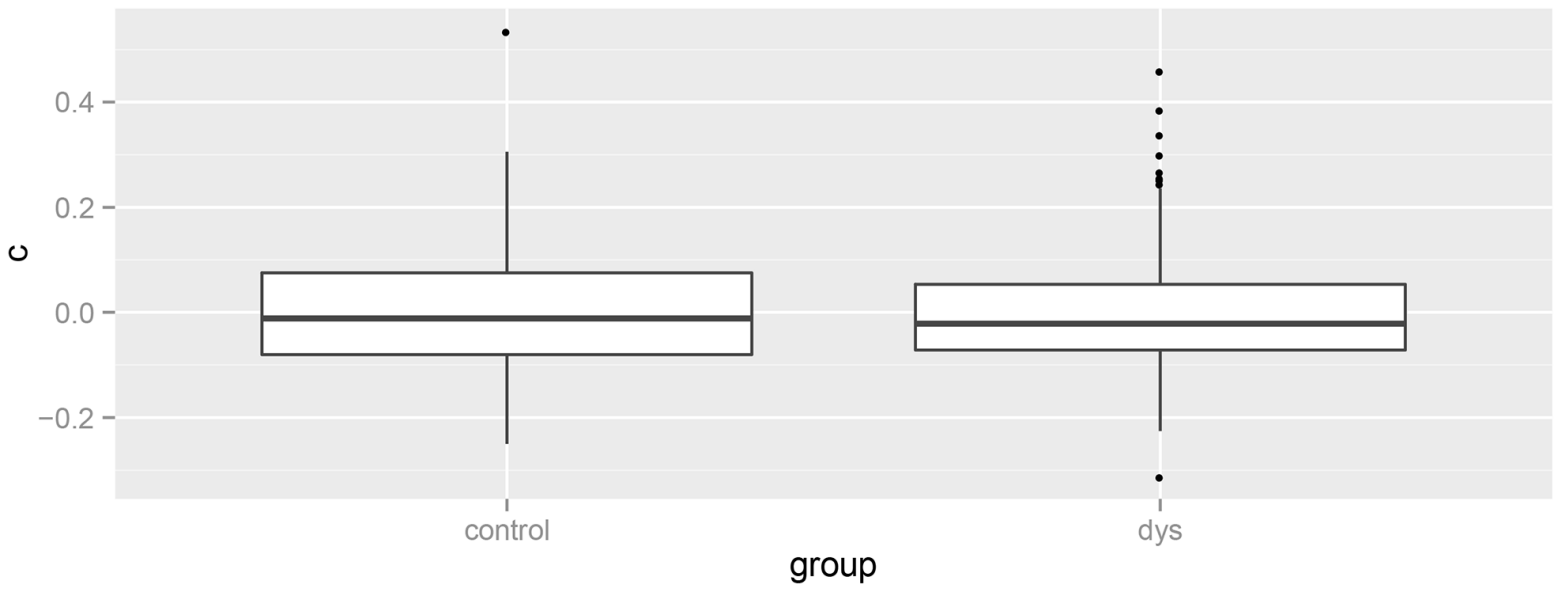
Boxplots of the subject-specific deviations of the response criteria, showing that there was no more variability in c1 scores of the students with dyslexia than in those of the age matched controls.

A possible objection against the above interpretation is that, although as a group the students with dyslexia did not perform worse, there were very large individual differences with some individuals with dyslexia having very negative c1-values and others having very positive c1-values. This would be reflected in the random intercepts (i.e., the subject-specific deviations of the response criteria). Figure 2 shows a boxplot of the random intercepts in both groups. The variability is not substantially larger in the group of students with dyslexia; it is even slightly smaller.

Table 2 further shows that the values of c2 in the dictation task were rather high (i.e., students needed quite high evidence for a spelling error before they said they were uncertain about their response). Again, however, the similarity between the students with dyslexia and the control students is larger than the difference between the groups.

Very much the same conclusions apply to the Proofreading task, although here both groups tended to be a bit too fast in their transition from certainly correct to rather certain correct (i.e., they put c1 at a level lower than warranted by their spelling proficiency). More importantly, however, there again was no obvious difference between the control group and the dyslexic group, as would be predicted by the Dual Burden theory.

## Discussion

The present study assessed whether undergraduate students in higher education suffer from bad metacognitive skills, as suggested by the dual burden theory [37], [38]. According to this theory, spelling errors in students with dyslexia are not only due to low proficiency levels (which cannot easily be changed in an individual), but also to the fact that students with dyslexia fail to realize when they have made a mistake (and hence cannot learn from their errors).

To examine the issue, we ran two tests with Feeling of Confidence (FOC) judgments. According to the dual burden theory, one would expect the difference between students with dyslexia and controls to be larger for scores that take FOC into account than for scores without FOC. However, we searched for an alternative way of analysis, which would give us much more information about the underlying processes. Signal detection is known to be the best framework to model decisions under uncertainty. Such an analysis allows researchers to distinguish criterion setting (which FOC is) from spelling proficiency. Due to recent statistical developments Signal Detection Analyses have become possible for many more designs than the psychophysical experiments on the basis of which they were originally developed.

The signal detection analysis of our data clearly confirmed the difference in proficiency between both groups (d′) and at the same time told us at which positions participants decided to say they were almost certain of their response or not certain at all anymore. As it turned out, the differences in criterion setting were not that much different between students with dyslexia and controls and most certainly not indicative of less rational strategies in students with dyslexia than in other students. Both groups of students seemed to be very smart in their criterion setting. As a result, we feel not justified to say that students with dyslexia in higher education suffer from a double burden (the situation may be different in primary school; this remains to be examined).

At the same time, the signal detection analysis allows us to evaluate the quality of the tests we used and to suggest improvements. For a start, it does not seem that the two-criterion response (certain, almost certain, uncertain) adds much to a single criterion response (certain: yes/no). In the present study, participants clearly put their most important criterion between certain and almost certain; c1). Only when it was clear that their spelling (or spelling correction) did not make sense, did they use the uncertain alternative.

Second, the dictation task also gave clearer data than the proofreading task. One reason for this may be the fact that participants could zoom in on non-target words in the sentence, thinking these were wrong (although they were not). It might be better in the future to alert the participants to the target words (e.g., by printing them in bold) and ask whether this part of the sentences is correctly spelled: yes or no (and how sure the participants are about their correction: sure vs. not sure). These designs will also be more straightforward to analyze. It would, however, require the addition of sentences in which the target words were spelled correctly.

All in all, we conclude that when signal detection theory analysis is used, there is no evidence for a dual burden in students with dyslexia in higher education. Their metacognitive skills are as good as they can be, given the fact that these students are making more mistakes because of their lower proficiency level. Remediation programs focusing on the metacognitive skill of FOC, therefore, are bound to fail. They may even do more bad than good, if the criterion setting is based on implicit learning rather than explicit, declarative knowledge [51].

Finally, because of the added value of the signal detection analysis and because this analysis may be rather daunting for someone starting with it (it took us quite a bit of time too), we include our data and the R program used to analyze them in the supplementary materials. In that way, everyone can first check whether they know how to do the analyses (by comparing their output with our data to Table 3), before they start to analyze other data.

We are convinced that these new types of analysis, although a little bit more complicated to apply, give a better picture of the true metacognitive qualities of people with more limited cognitive skills. After we finished data collection, we discovered another study of Maniscalco and Lau [52] that takes the same approach. These authors employed a new method derived from classical SDT to isolate metacognitive evaluation from task performance on the basis of correct and incorrect decisions. They argue for the use of a relative sensitivity measure instead of an absolute sensitivity measure. Relative sensitivity makes it possible to separate the quality of the information being metacognitively evaluated from the quality of the metacognitive evaluation itself. This (new) type of sensitivity, called *meta-d′*, is another interesting measure to reveal the efficacy of a person's metacognitive experiences.

## Supporting Information

File S1 **This is a text file with the raw data.** For each participant and each trial of the dictation and the proofreading task, it says how certain the participant felt and whether the answer was correct or not.
(TSV)

File S2 **This is a file to run the statistical analyses in R.** Once you are in the R programming environment, it should run on the data file (if the latter has been placed in your working directory). You must make sure that the package Ordinal is loaded as well. The output of the analyses can directly be compared to the summary in Table 3.
(R) Click here for additional data file.
